# Preoperative Synovial Fluid Cultures, and Biopsy Cultures After Dry Tap Aspiration, Are Valuable in Diagnosing a Periprosthetic Joint Infection: A Retrospective Study

**DOI:** 10.3390/microorganisms13030562

**Published:** 2025-03-01

**Authors:** Bart Copier, David Visser, Jakob van Oldenrijk, Pieter Koen Bos, Ewout S. Veltman

**Affiliations:** Department of Orthopaedic Surgery and Sports Medicine, Erasmus MC, 3015 GD Rotterdam, The Netherlands; b.copier@erasmusmc.nl (B.C.); d.visser@erasmusmc.nl (D.V.); j.vanoldenrijk@erasmusmc.nl (J.v.O.); p.k.bos@erasmusmc.nl (P.K.B.)

**Keywords:** periprosthetic joint infection, total hip arthroplasty, synovial fluid aspiration, dry tap aspiration, tissue biopsy

## Abstract

Periprosthetic joint infection (PJI) is a devastating complication after total hip arthroplasty. Synovial fluid aspiration and preoperative tissue biopsy cultures can be helpful diagnostic tools for PJI. The aim of this study is to evaluate the diagnostic value of synovial fluid aspirations in general, and preoperative biopsies after inconclusive or dry tap aspiration in patients undergoing revision hip arthroplasty in particular. Patients who underwent diagnostic aspiration and subsequent preoperative biopsy and/or revision surgery between January 2015 and January 2024 were included in the study. Synovial fluid aspirations and tissue samples obtained from biopsy and revision surgery were interpreted using the European Bone and Joint Infection Society criteria for PJI. In total, 207 patients were included with 231 synovial fluid aspirations. The sensitivity and specificity of synovial fluid aspiration cultures were 76% and 98%. In 62 cases, tissue biopsies were performed, of which 23 were after dry tap aspiration. Tissue biopsies after dry tap aspiration had a sensitivity of 44% and a specificity of 93%. Tissue biopsies after dry tap aspiration led to the confirmation of PJI in 7/23 cases. Synovial fluid aspiration yields reliable results when evaluating a patient for suspicion of PJI. Diagnosing PJI can, however, be troublesome if the synovial fluid aspiration provides a dry tap or inconclusive result. Tissue biopsy cultures after dry tap aspiration are a feasible way to confirm PJI.

## 1. Introduction

Periprosthetic joint infection (PJI) after total hip arthroplasty (THA) occurs in approximately 1–2% of patients [1]. PJI is one of the major reasons for revision after THA [2]. PJI is a devastating complication and is associated with lower quality of life and higher mortality compared to patients without PJI [3]. Diagnosing PJI is challenging, because there is no gold standard diagnostic test [4]. The European Bone and Joint Infection Society (EBJIS) has developed diagnostic criteria for PJI, which were updated in 2021 [5,6]. Diagnosing PJI prior to revision arthroplasty is essential to determine an adequate treatment strategy [7].

Synovial fluid aspiration can be helpful to diagnose PJI. The sensitivity of synovial fluid aspiration cultures is relatively low [8]. Therefore, a negative fluid aspiration culture cannot always reliably rule out PJI. The sensitivity is especially low in patients with a low-grade PJI [9]. Synovial fluid cultures can also yield contradictory results; a combination of a normal leukocyte count with a positive culture or an abnormal leukocyte count with a negative culture result can be difficult to interpret. Synovial fluid aspiration results, therefore, can be inconclusive.

Synovial fluid is not always present in quantities enabling aspiration or can just be missed with the needle, which leads to dry tap aspiration. A dry tap occurs in approximately 36% of attempted hip aspirations [10]. A dry tap aspiration does not rule out PJI [11]. Open biopsies can be performed after dry tap aspiration or inconclusive synovial fluid culture results.

The aim of this study is to evaluate the diagnostic accuracy of synovial fluid aspiration cultures in general and to specifically evaluate the clinical value of preoperative tissue biopsies after inconclusive or dry tap aspiration of the hip in patients undergoing revision hip arthroplasty for both septic and aseptic pathologies.

## 2. Methods

### 2.1. Study Design

This observational study was approved by the local medical ethics board. We retrospectively constructed a database from the biweekly arthrocentesis outpatient clinic. We used electronic patients records (HiX 6.2) to identify eligible participants. We selected patients if a sterile synovial fluid aspiration of the hip was performed between January 2015 and January 2024 for the diagnosis of PJI. Patients who underwent diagnostic aspiration and/or subsequent preoperative tissue biopsy and/or revision surgery were included in the study. Patients were excluded if aspiration results were not available and/or if no tissue cultures were obtained during revision surgery.

We extracted patient data such as demographic characteristics, comorbidities and orthopedic history. The results of infection diagnostics were extracted. Serum Leukocyte count (WBC) and C-reactive protein (CRP) were determined. A leukocyte count was performed on the synovial fluid obtained during hip aspiration. The percentage of polymorphonuclear neutrophils (PMNs) was determined. If there was a fistula or wound leakage at the time of outpatient clinic assessment, this was reported. The results of the synovial fluid aspiration cultures, preoperative tissue biopsies, intraoperative tissue samples and sonication fluid cultures were reported. Radiological imaging results were collected and analyzed for signs of loosening or osteolysis, in patients in whom a current Girdlestone radiological analysis was not performed.

### 2.2. Synovial Fluid Aspirations

Synovial fluid aspirations were carried out in sterile conditions in a specialized biweekly outpatient clinic. Before the synovial fluid aspiration was performed, the correct position for needle introduction was determined under fluoroscopy. The skin was disinfected with chlorhexidine. After insertion of the needle, the position in the joint was confirmed with fluoroscopy. Depending on the amount of synovial fluid aspirated, it was distributed among a Port-A-Cul specimen transport tube (Henry Schein Medical, Almere, Holland) an anaerobic (lytic/10 anaerobic/F BD BACTEC^TM^) and aerobic (plus aerobic/F BD BACTEC^TM^) blood culture bottle and an EDTA tube (BD vacutainer K2EDTA 7.2 mg). The obtained material was then sent for culture analysis and/or leukocyte count. If no synovial fluid was aspirated (dry tap), the joint could be flushed with 5–10 cc sterile 0.9% NaCl. The fluid obtained after flushing was then sent for culture analysis. The microbiology results of these synovial fluid aspiration cultures were not analyzed separately, but as part of the entire cohort. Not in all cases of dry tap was 5–10 cc sterile 0.9% NaCl introduced as the protocol changed during the study period. Synovial fluid aspiration results were considered positive if the culture became positive and/or leukocyte count was higher than 3000 × 10^6^/L and if more than 80% were PMNs, adhering to the EBJIS diagnostic criteria for PJI [6]. A combination of a normal leukocyte count with a positive culture and an abnormal leukocyte count with a negative culture result were deemed as an inconclusive aspiration result outcome.

### 2.3. Tissue Cultures

In case of dry tap, but high clinical suspicion of PJI, tissue biopsies were taken. This suspicion was based on clinical, laboratory or radiological findings in combination with patient history, as assessed by the treating orthopedic infection specialist. Biopsies could also be taken after an inconclusive synovial fluid aspiration result. Biopsies were taken during a percutaneous procedure in the operating room with the patient under general anesthesia. After the skin was disinfected and the correct position was marked, 4 to 6 tissue and/or bone samples from the implant bone interface area were obtained with Jamshidi needles through a stab incision under fluoroscopic guidance. The obtained tissues were sent to the microbiology laboratory for analysis.

During revision surgery per protocol, at least 4 tissue samples and prosthesis components were taken and placed in sterile containers. After sonication, both tissue samples and sonication fluid of the prothesis were sent for culture to the medical microbiology department. Intraoperative tissue cultures in combination with the sonication fluid culture were considered positive when two cultures became positive with the same microorganism. In case of one positive culture this was regarded as contamination in close consultation with the microbiologist. The same criteria were used for the tissue biopsy cultures. Fluid and tissue samples were incubated for at least 14 days before they were declared negative [12]. Per protocol, no antibiotics were prescribed for at least 2 weeks before biopsy or revision surgery, to prevent false negative results as previously described by Spangehl et al. [13]. If antibiotics were prescribed prior to tissue biopsy or revision surgery, this was reported. The final PJI diagnosis was established in close consultation with the microbiologist in a multidisciplinary infection meeting and adhering to the EBJIS diagnostic criteria for PJI [6]. This final diagnosis was retrieved from the digital patient record and registered in our database.

### 2.4. Outcome Measures

The final evaluation of the EBJIS diagnostic criteria for PJI, as assessed by the multidisciplinary infection meeting, was considered as the gold standard. The outcome of the synovial fluid aspiration culture, preoperative tissue biopsy cultures and/or intraoperative tissue cultures in combination with the sonication fluid culture were compared with the gold standard. Patients in whom, after inconclusive synovial fluid aspiration, a preoperative biopsy was subsequently performed, were analyzed separately and as a part of the entire cohort. We also evaluated in which part of the patients the addition of tissue biopsies following aspiration of the hip caused a change in treatment.

### 2.5. Statistics

Based on the number of true positives, false positives, true negatives and false negatives, the sensitivity and specificity of the synovial fluid aspiration cultures, tissue biopsy cultures and intraoperative tissue cultures can be calculated. Subsequently, the positive predictive value (PPV) and negative predictive value (NPV) can be determined. Sensitivity, specificity, PPV and NPV were calculated with a 95% confidence interval. All analyses were performed in IBM SPSS Statistics (version 28.0.1.0, Windows).

## 3. Results

Between January 2015 and January 2024, we included 207 patients with 231 synovial fluid aspirations of the hip. The demographic characteristics of the included cases are shown in Table 1. Twelve patients had synovial fluid aspiration within three months of onset of symptoms. These patients were considered suspected acute infections; all other patients had chronic symptoms. Revision surgery was performed in 212/231 cases. In 19 cases, tissue biopsy tissue cultures were taken, but no revision THA was performed.

In 197/231 cases, synovial fluid aspiration provided diagnostic information. In 193 cases, synovial fluid aspiration cultures were available. In four cases, synovial fluid white blood cell count and % PMN were determined but no synovial fluid cultures were taken.

**Figure 1:**
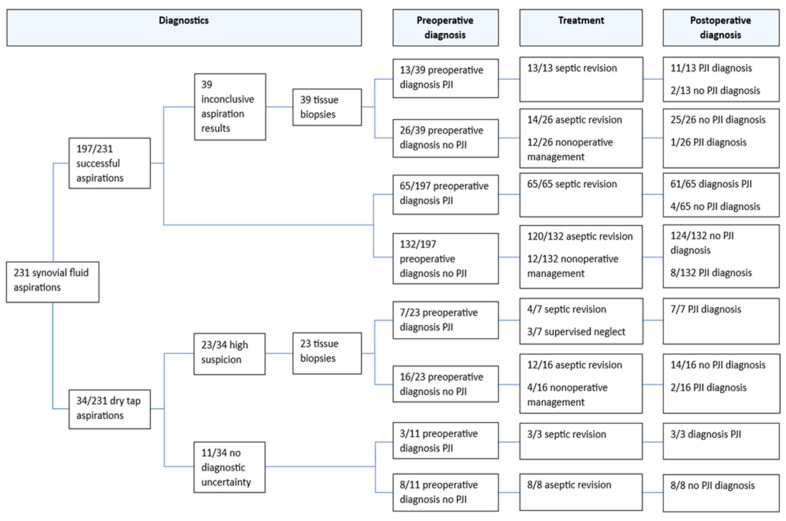
Flowchart. PJI, periprosthetic joint infection.

In 40/193 (21%) cases, aspiration cultures were obtained after flushing the hip joint with NaCl after an initial dry tap aspiration. In 34 cases, synovial fluid aspiration resulted in a dry tap (Figure 1). Synovial fluid aspiration cultures had a sensitivity of 76% (95% CI, 65–85%) and a specificity of 98% (95% CI, 94–100%) for diagnosing PJI. In 81 cases, the number of leukocytes and the percentage of PMN leukocytes in the synovial fluid were determined. The sensitivity, specificity and predictive values are shown in Table 2.

Tissue biopsy cultures were obtained in 62 cases. The sensitivity, specificity and predictive values of all 62 tissue biopsy cultures are shown in Table 2. In 23 cases, biopsies were taken after dry tap aspiration, and in 39 cases because of an inconclusive aspiration result outcome. In 16/23 dry tap cases, subsequent revision surgery was performed. We found a sensitivity and specificity of 44% (95% CI, 17–75%) and 93% (95% CI, 72–100%), for tissue biopsy cultures after dry tap aspiration, Table 3. In 4/23 cases, tissue biopsy cultures confirmed the clinical suspicion of PJI and changed the treatment strategy. In two of these four cases, septic two-stage revision arthroplasty was performed and in two cases supervised neglect was chosen as the treatment strategy.

Thirty-nine biopsies were taken after an inconclusive aspiration results outcome. Tissue biopsy cultures after an inconclusive synovial fluid aspiration led to a change in the diagnostic result in 7/39 cases. In 4/7 cases, tissue biopsy cultures confirmed PJI after a previous negative synovial fluid aspiration; these cases were considered septic and treated as such. In 3/7 cases, tissue biopsy cultures were negative after a previous positive synovial fluid aspiration. These cases were considered aseptic and were treated as such.

Culture results can be found in Table 4. Similar microorganisms were found in synovial fluid aspirations and intraoperative tissue cultures, while the tissue biopsy cultures only yielded low-virulent pathogens. The most common microorganisms were coagulase-negative staphylococci. The same causative pathogens were found in all tissue biopsy cultures and in the intraoperative tissue cultures.

In 212/231 cases, intraoperative tissue cultures and sonication fluid cultures were available. The sensitivity, specificity and predictive values are shown in Table 2. In 3/13 cases with a false negative result, antibiotics were prescribed within two weeks prior to surgery. In 19 patients, after informed consent, a nonoperative treatment was chosen, and revision tissue cultures are therefore not available.

## 4. Discussion

This study evaluates the diagnostic value of synovial fluid aspirations, and the addition of preoperative biopsies after inconclusive aspiration results or dry tap aspiration of the hip prior to revision THA in patients with a clinical suspicion of PJI. The sensitivity and specificity of biopsies after dry tap aspiration were 44% and 93%. The corresponding PPV and NPV were 80% and 72%. In 16/23 cases with a suspected PJI, tissue biopsy cultures refuted the PJI suspicion after dry tap aspiration and subsequently directly changed the patients’ treatment to an aseptic regimen. In 7/23 patients, PJI was confirmed (Figure 1).

In addition, better diagnostic information, by identifying the causative microorganism and its antibiotic sensitivity through a tissue biopsy, can lead to a well-informed decision between one- and two-stage revision surgery as a treatment strategy [14]. A septic one-stage revision may lead to improved functional outcomes compared to a two-stage approach [15,16]. In 7/39 cases, additional tissue biopsy cultures led to a change in treatment strategy after an inconclusive synovial fluid aspiration. The change in treatment strategy concerned both aseptic-to-septic revision and vice versa.

Studies by Fink et al. found a sensitivity and specificity of 82–94% and 94–98% for preoperative biopsies prior to revision surgery [17,18]. In these studies, a preoperative biopsy was performed in every revision case, not only in cases with dry tap synovial fluid aspiration. A study by Sconfienza et al. describes the diagnostic value of preoperative biopsies after dry tap aspiration. They performed ultrasound-guided, percutaneous periprosthetic biopsies in 40 patients after a dry tap aspiration. A sensitivity and specificity of 42% and 100% was found [19]. However, during the biopsy procedure in this study, only one culture was taken. A study by Ottink et al. describes the diagnostic value of biopsies obtained in the operating room with a thick-bore needle in patients with a suspected chronic PJI. Twenty-nine tissue biopsies were included which led to a sensitivity and specificity of 82% and 100%. In the study by Ottink et al., tissue cultures obtained during revision surgery were used as the gold standard, which differs from our study [20]. This may explain the difference in sensitivity between the studies. Simon et al. described the diagnostic value of biopsies after a negative joint aspiration culture or a dry tap in patients with suspected low-grade PJI, and found a sensitivity of 80% and a specificity of 69% [21]. Their conclusion was that biopsies have a limited predictive value for diagnosing PJI. However, there were only six cases with dry tap aspiration and the authors fail to notice the percentage of patients in which the addition of tissue biopsy cultures caused a clinically relevant change of treatment.

Tissue biopsy cultures had a low sensitivity. The use of antibiotics prior to a preoperative biopsy is a common reason for a false negative result [22]. Tissue cultures can be unable to reliably detect microorganisms that are embedded within a biofilm, which protects the infecting organisms from being recovered. Additionally, conventional cultures are frequently unable to grow low-virulent pathogens or fungi, leading to more false negative results [23]. Tissue biopsy cultures only detected low-virulent bacteria (Table 4). In our study, cases where antibiotics were prescribed within 2 weeks of culture collection were not excluded. This leads to lower sensitivity but approximates clinical practice.

In 39 cases, a biopsy was performed after a successful synovial fluid aspiration with an inconclusive result. These biopsies were performed when there was a discrepancy between the initial clinical suspicion of PJI and the aspiration results. In our study, biopsy cultures after an inconclusive joint aspiration changed the preoperative diagnosis in 7/39 cases. Hassebrock et al. described the role of repeat synovial fluid aspiration for diagnosing PJI. They found that a second synovial fluid aspiration additionally diagnosed PJI in 6/30 cases and ruled out PJI in 2/30 cases. Thus, a second joint aspiration accurately changed the preoperative diagnosis in 8/60 cases, which is lower than our findings [24]. Furthermore, in the study by Hassebrock et al., a definitive preoperative diagnosis could not be made in 25/60 cases, based on the results of the joint aspirations [24]. This is because only one culture can be obtained during hip aspiration and there is not always enough synovial fluid to perform all diagnostic tests. This suggests that subsequently taking tissue biopsies is a better option than repeat hip aspiration for diagnosing PJI in this specific group.

The sensitivity and specificity of synovial fluid aspiration cultures in our study were 76% and 98%. These results are in line with previous studies [8,25]. The meta-analysis of Qu et al. found a pooled sensitivity and a specificity of 70% and 94% [25]. Similar results were found in the meta-analysis by Carli et al. [8]. In 40/193 procedures, joint aspiration cultures were obtained after flushing the hip joint with sterile NaCl. The diagnostic value of cultures obtained after flushing the hip with NaCl is controversial. In the literature, a high sensitivity and specificity is described [26,27]. However, some studies did find a lower sensitivity when joint aspiration cultures were diluted with NaCl [10,28]. Since our results are consistent with the literature, we believe that the cultures obtained after flushing the hip joint with NaCl did not alter our results.

Synovial fluid analysis includes a leukocyte count and a calculation of the percentage of polymorphonuclear neutrophils (PMNs). A high number of leukocytes and a high percentage of PMNs (>3000 and >80% respectively according to EBJIS criteria) would be confirmatory of PJI [6]. In our study, the results were only available in 81 patients, as the others had either a dry tap or an insufficient amount of synovial fluid for chemical analysis. The synovial fluid leukocyte count can introduce confusion, as there are other factors such as wear, metallosis, and rheumatologic disorders such as gout or pseudogout can cause a high synovial leukocyte count. Therefore, in case of a low-volume successful joint aspiration, we prefer to use it for culture rather than chemical analysis.

In recent years, several new diagnostic tools have become commercially available for the detection of PJI. Even though the results are promising, wide-spread adoption is still limited due to the relatively high costs of these tests [29]. Molecular techniques such as polymerase chain reaction (PCR) and next-generation sequencing may be valuable additional diagnostics to detect PJI [30]. A meta-analysis by Cheng Li et al. shows that PCR of synovial fluid leads to a sensitivity of 70%, which is comparable to synovial fluid cultures [31]. However, standard cultures do have limitations compared to PCR. PCR is superior when it comes to identifying low-virulence microorganisms, PCR results are available faster compared to cultures and PCR is still accurate after previous antibiotic treatment [32,33]. The advantages of PCR become clear in the study by Ghirardelli et al. In this study, PCR was the main determinant for diagnosing PJI and identified the causative microorganism in 63% of patients, versus 26% with standard cultures [33].

Next-generation sequencing (NGS) is an upcoming technology with the ability to identify microorganisms in synovial fluid. A meta-analysis of Hantouly et al. shows that NGS has an excellent sensitivity of 94%, which is a lot higher than the sensitivity of 70% for synovial fluid cultures. However, the specificity of NGS for diagnosing PJI was lower compared to cultures, 89% versus 94% [34]. A high sensitivity is valuable in case of hard-to-detect low-virulent pathogens. This is clearly shown in the study by Hong et al. In this study, NGS identified potential causative microorganisms in 47/98 (48%) cases with a culture-negative PJI [29]. In a smaller study with 11 cases with a culture negative PJI, NGS was able to identify a potential microorganism in up to 82% [35]. An important limitation of NGS is the possibility of false positive results, since it is difficult to differentiate between contamination and infection. This leads to a lower specificity [34,36].

Molecular biologic tests can be incorporated into daily practice in a variety of ways. They could be implemented as a standard part of the analysis for PJI. However, as there are considerable costs related to the use of these tests, and the current diagnostic workflow already achieves acceptable diagnostic accuracy, this is considered an unnecessary expenditure. It may be more feasible to introduce molecular biologic tests in cases of diagnostic uncertainty, such as in the group of patients with an inconclusive aspiration or dry tap. In that case, it would be an option to include these tests in combination with the tissue cultures during biopsy. Finally, one could introduce the molecular biologic test as an additional diagnostic during revision surgery in the cases where uncertainty remains even after biopsy culture results. In this case, it would be needed in the least number of cases and therefore would introduce lower costs. Which of these different integrations in daily care is cost-efficient and provides the most benefit to the patient should be studied prospectively.

The limitations of this study are reflective of its retrospective design. In some cases, information was incomplete or unclearly written in medical records and, therefore, not all variables were available. Histological findings were available in few cases; therefore, these were not evaluated even though they are part of the EBJIS criteria. Our study lacks a direct comparison between synovial fluid aspiration cultures and biopsy cultures. We have not made the direct comparison, because we feel the most important incentive to consider performing biopsy is a dry tap aspiration. As in the case of dry tap there is no result to compare to the biopsy result, and for inconclusive results the decision to perform additional biopsy is subjective, we feel the retrospective design of our study may introduce bias in a direct comparison. Direct comparison of synovial fluid aspirations and tissue biopsy cultures could be the subject of a prospective evaluation. Finally, not in all cases was revision surgery performed. Thus, the final PJI diagnosis has not been confirmed with intraoperatively taken tissue cultures in all cases.

In this study, we did not perform a formal cost-effectiveness analysis of the diagnostic work-up. However, we can speculate on whether adding a biopsy after dry tap aspiration of the hip is cost-effective. The costs of biopsies are relatively low. They are performed in the OR under general anesthesia, but patients are admitted and discharged on the same day, and estimated costs are EUR 500–700 including the costs for the cultures. The costs for a two-stage revision are conservatively estimated at around EUR 30,000–50,000, the costs for aseptic (partial) revision at EUR 6000–8000 if complications are avoided. Therefore (in the worst-case scenario), the number needed to treat to be cost-effective would be around 31 biopsies ((30,000 − 8000)/700 = 31.35). Probably, the numbers are even more favorable.

Many studies on the value of synovial fluid aspiration, additional biopsy and peroperative tissue cultures solely focus on the measured sensitivity and specificity of these diagnostic procedures. A low sensitivity of tissue biopsy cultures can easily be misinterpreted as a reason not to perform a biopsy even when clinical suspicion of PJI is present. Our study evaluates the percentage of cases in which clinically relevant changes to the treatment strategy were caused by the tissue biopsy results. For further research, our findings need to be validated in a larger prospective study.

## 5. Conclusions

Synovial fluid aspiration yields reliable results when evaluating a patient for suspicion of PJI. Tissue biopsy cultures in patients with a high suspicion of PJI after dry tap or inconclusive aspiration are a feasible way to confirm PJI. Ruling out PJI by means of a biopsy after a dry tap aspiration is less successful due to its low sensitivity. We recommend obtaining tissue biopsies in patients with a high suspicion of PJI, but dry tap aspiration. Negative test results should be interpreted with caution, given the risk of a false negative result. In patients with an inconclusive aspiration, taking tissue biopsy cultures can be considered instead of repeat joint aspiration for diagnosing PJI; whether this is mandatory should be evaluated prospectively.

## Figures and Tables

**Table 1:** Demographic characteristics.

Demographics	Synovial Fluid Aspiration (n = 231)	Tissue Biopsy (n = 62)
Mean age (SD)	65.4 (12.4)	62.7 (11.8)
Female (%)	125 (54.1)	36 (58.1)
ASA (%)		
1	15 (6.5)	6 (9.7)
2	106 (45.9)	30 (48.4)
3	103 (44.6)	23 (37.1)
4	7 (3.0)	3 (4.8)
Mean BMI (SD)	28.7 (5.6)	29.3 (6.1)
Revision surgery (%)	212/231	43/62
Aseptic revision	106 (50.0)	19 (44.2)
Septic revision	106 (50.0)	24 (55.8)
Reimplantation after girdlestone	34/106 (32.0)	7/24 (29.2)
Revision THA (%)	96 (41.6)	19 (30.6)
History of PJI (%)	77 (33.3)	20 (32.3)
Median Leukocytes (IQR)	7.7 (6.4–9.4)	7.9 (7.0–9.1)
Median CRP (IQR)	7.4 (2.3–20)	8.4 (2.9–22)
Radiological loosening (%)	130/196 (66.3)	32/54 (51.6)

THA, total hip arthroplasty; PJI, periprosthetic joint infection.

**Table 2:** Distribution of results of joint aspirations, tissue biopsy cultures and intraoperative tissue cultures.

	Synovial Fluid Aspiration Cultures	Synovial Fluid White Blood Cell Count and % PMN	Tissue Biopsy Cultures	Intraoperative Tissue Cultures
True positives	51	18	11	65
True negatives	123	55	40	133
False positives	3	4	1	1
False negatives	16	4	10	13
Total	193	81	62	212
Sensitivity (95% CI)	76.1 (65–85%)	81.8 (63–94%)	52.4 (32–73%)	83.3 (74–90%)
Specificity (95% CI)	97.6 (94–99%)	93.2 (85–98%)	97.6 (90–100%)	99.3 (97–100%)
PPV (95% CI)	94.4 (86–99%)	81.8 (63–94%)	91.7 (68–100%)	98.5 (94–100%)
NPV (95% CI)	88.5 (83–93%)	93.2 (85–98%)	80.0 (68–89%)	91.1 (86–95%)

PMN, polymorphonuclear neutrophils; PPV, positive predictive value; NPV, negative predictive value.

**Table 3:** Tissue biopsy culture results after dry tap aspirations.

	Tissue Biopsy Cultures
True positives	4
True negatives	13
False positives	1
False negatives	5
Total	23
Sensitivity	44.4 (17–75%)
Specificity	92.9 (72–100%)
PPV	80.0 (37–99%)
NPV	72.2 (50–89%)

PPV, positive predictive value; NPV, negative predictive value.

**Table 4:** Microorganisms found in synovial fluid aspirations, tissue biopsy cultures and intraoperative cultures.

	Synovial Fluid Aspirations	Tissue Biopsy Cultures	Intraoperative Cultures
Coagulase-negative staphylococci	27	9	34
staphylococcus aureus	4	0	6
Enterobacteriaceae	5	0	6
Streptococcus species	4	4	7
Enterococcus species	4	0	7
Cutibacterium acnes	3	1	8
Coryneform bacteria	1	1	3
Others	6	0	7
Polymicrobial	0	2	8

## Data Availability

The original contributions presented in this study are included in the article. Further inquiries can be directed to the corresponding author.
